# Postoperative Acute Exacerbation of IPF after Lung Resection for Primary Lung Cancer

**DOI:** 10.1155/2011/960316

**Published:** 2011-02-07

**Authors:** Atsushi Watanabe, Nobuyoshi Kawaharada, Tetsuya Higami

**Affiliations:** Department of Thoracic and Cardiovascular Surgery, Sapporo Medical University School of Medicine, South 1, West 16, Chuo-ku, Sapporo 060-8543, Japan

## Abstract

Idiopathic pulmonary fibrosis (IPF) is characterized by slowly progressive respiratory dysfunction. Nevertheless, some IPF patients experience acute exacerbations generally characterized by suddenly worsening and fatal respiratory failure with new lung opacities and pathological lesions of diffuse alveolar damage. Acute exacerbation of idiopathic pulmonary fibrosis (AEIPF) is a fatal disorder defined by rapid deterioration of IPF. The condition sometimes occurs in patients who underwent lung resection for primary lung cancer in the acute and subacute postoperative phases. The exact etiology and pathogenesis remain unknown, but the condition is characterized by diffuse alveolar damage superimposed on a background of IPF that probably occurs as a result of a massive lung injury due to some unknown factors. This systematic review shows that the outcome, however, is poor, with postoperative mortality ranging from 33.3% to 100%. In this paper, the etiology, risk factors, pathogenesis, therapy, prognosis, and predictors of postoperative AEIPF are described.

## 1. Introduction and Background

The incidence of lung cancer in patients with idiopathic pulmonary fibrosis (IPF) is higher than the general population, with relative risks reported to be from 7 to 14% [1–3] IPF is usually a gradually progressive but an ultimately fatal disease. Although the disease is chronic in nature, abrupt worsening can occur in some patients. This condition was first introduced by Kondoh and Saiki [4] and was then called acute exacerbation of IPF (AEIPF). The prognosis of AEIPF is usually considered to be grave, but it has been reported that some patients show improvement following corticosteroid therapy. It still remains uncertain what causes such an acute exacerbation, and appropriate therapy for this condition has not been established. In the survey by the Japanese association of thoracic surgery, 1036 of 27881 patients who underwent pulmonary resection for primary lung cancer during the year 2008 had interstitial pneumonia as a preoperative comorbidity. Although the hospital mortality was about 0.9% (248 patients died after the operation), 63 of 248 patients (25.4%) died of interstitial pneumonia, including AEIP. We are focused on the current knowledge of AEIPF and what causes the exacerbation after pulmonary resection for nonsmall cell lung cancer (NSCLC).

## 2. The Content

### 2.1. Diagnostic Criteria for Acute Exacerbation of IPF

The acute exacerbation of IPF (AEIPF) is characterized by diffuse and rapid alveolar damage superimposed on a background of IPF that probably occurs as a result of a massive lung injury due to some unknown etiologic agent. The definition of AEIPF was first described by Yoshimura et al. [5]. The characteristics include (1) intensified dyspnea, (2) increase in the interstitial shadow on chest radiograph, (3) increase in fine crackles on auscultation, (4) elevation of serum lactate dehydrogenase, and (5) decrease in arterial oxygen tension of more than 10 mm Hg under similar condition. After then, some diagnostic criteria have been described [6–11]. In the clinical field and the surgical field, the definition described by Hyzy has been generally applied (Table 1).

Especially, bacterial pneumonia must be distinguished from AEIPF. Pneumonia is diagnosed by the presence of new and/or progressive pulmonary infiltrates on chest radiography plus two or more of the following criteria: fever (38°C), leukocytosis (12 × 109/L), purulent sputum, or isolation of pathogen in respiratory secretions. If necessary, endotracheal aspiration or BA is performed.

### 2.2. Etiology

Specific factors causing AEIPF have not been elucidated. However, some cases of AEIPF have occurred after lung resection or biopsy [13, 21]. Kondoh et al. [22] observed that postbiopsy exacerbation occurred in 2.1% of 236 consecutive patients who underwent surgical biopsy for diffuse lung disease. AEIPF appear to occur at any time during the course of disease and may be the presenting manifestation for some patients. Importantly, the risk of an exacerbation does not appear to be linked to the level of pulmonary function [23]; although in one prospective series, patients with lower forced vital capacity had more total and respiratory-related hospitalizations during subsequent followup [24]. There is no clear association with age or smoking history, but acute exacerbations seem to be more common in men.

### 2.3. Pathology

AEIPF is an acute insult to the lung over a background of IPF. According to some autopsy studies [25–27], there was a wide distribution in the extent of fibroblastic foci but not seen in fibrotic nonspecific interstitial pneumonia. Fibroblastic foci were distinguished from buds of intra-alveolar organization seen in the organizing phase of diffuse alveolar damage by their immediate adjacency to areas of established fibrosis and their presence away from areas of established diffuse alveolar damage. Areas showing honeycomb changes contained aggregates of abundant neutrophils within the air spaces; however, staining for organisms was uniformly negative in these areas and the results for tissue taken for culture at autopsy also were negative.

### 2.4. Incidence and Mortality of Postoperative AEIPF

According to the annual report by the Japanese association of thoracic surgery [28], 1036 of 27881 patients (3.7%) who underwent pulmonary resection for primary lung cancer during the year 2008 had interstitial pneumonia as a preoperative comorbidity. Although the whole hospital mortality was about 0.9% (248 patients died after the operation), 63 patients (25.4%) died of interstitial pneumonia, including AEIPF. Additionally, 157 patients suffered from AEIPF during hospitalization.

Six (15.0%) of 40 patients with IPF had AEIPF after the operation and 5 of the six died with respiratory failure at POD 47 on average (range 17–95 days) [20]. We reported that 4 (7.1%) of these 56 patients developed postoperative AEIPF, and all of them died of respiratory failure within 42 days after the operation despite immunosuppression with pulse doses of methylprednisolone [18]. The rate of occurrence and mortality of postoperative AEIPF in the literatures [12, 14, 16, 17] are summarized in Table 2. The incidence of postoperative AEIPF ranged from 0% to 20.8%, mortality after pulmonary resection from 8.3% to 22.9%, and mortality after occurrence of AEIPF from 37.5% to 100%. On the other hand, AEIPF may occur after any medical treatment for cancer. Minegishi et al. reported that the incidence and mortality after each anti-lung cancer therapy, including pulmonary resection, were 20% and 14% for chemotherapy (*n* = 50), 42.9% and 14.3% for chemoradiotherapy (*n* = 7), 16.7% and 16.7% for radiation therapy (*n* = 6), 22.9% and 8.9% for pulmonary resection (*n* = 35), and 31.3% and 31.3% for best supportive care (*n* = 32) [19], respectively.

### 2.5. Risks of Postoperative Exacerbation of IPF

Kumar et al. [13] reported that postoperative ARDS was associated with lower preoperative levels of carbon monoxide diffusion capacity corrected for alveolar volume (*K*_CO_), lower preoperative DLCO levels, and higher preoperative composite physiological index [29] (CPI = 91.0 − (0.65 ×  % predicted DLCO) − (0.53 ×  % predicted FVC) + (0.34 ×  % predicted FEV1)). A preoperative CPI score >40 was associated with a 50% chance of developing postoperative lung injury. The occurrence of ARDS was not related to demographic features, smoking history, other pulmonary function variables, presence of preoperative dyspnea, histologically established diagnosis of pulmonary fibrosis before pulmonary resection, stage of nonsmall cell lung cancer, or pattern of inflammation. 

% vital capacity (VC) (<80.6%) and LDH level (≥241 IU/L) achieved a complete classification of patients with AEIPF from those without AEIPF [28]. The other researchers reported that postoperative AEIP were associated with %VC < 80% [16, 17], low DLCO [16], and %TLC < 95% [29].

The incidence of postoperative complications associated with video-assisted thoracoscopic surgery (VATS) seemed to be low although the use of VATS could not prevent AE of usual interstitial pneumonia (UIP) [15].

On the other hand, our study demonstrated no observed predictive risks of postoperative AEIPF after lung resection for primary lung cancer [18]. The risks, which are reported in the literatures, are shown on Table 3. However, these reports are from the study of a single institution and the number of patients is too small for statistical analysis of the risks. The aforementioned pose as limitations of the study. In the Japanese association of chest surgery, a multi-institutional Study of the postoperative exacerbation of IPF is currently being conducted.

### 2.6. Strategy for Decreasing the Incidence of Postoperative AEIPF

Surgical approaches, such as conventional thoracotomy, muscle sparing thoracotomy, and video-assisted thoracoscopic surgery have no effect on the occurrence of postoperative AEIPF [30]. Some unknown or potential etiologic agents of AEIPF must be induced by pulmonary resection or some factors related to pulmonary resection, such as selective lung ventilation and manipulation of the ipsilateral lung. If oxygen radical toxicity can be associated to the occurrence of AEIPF, Misthos et al. [31] recently reported this interesting theory. The authors revealed the following results: (1) lung re-expansion after one-lung ventilation (OLV) provoked severe oxidative stress; (2) the degree of generated oxygen-derived free radicals was associated with the duration of OLV; (3) patients with lung cancer had a higher production of oxygen-derived free radicals than the normal population; (4) tumor resection removes a large oxidative burden from the organism; (5) mechanical ventilation and surgical trauma are weak free radical generators; (6) manipulated lung tissue is also a source of oxygen-derived free radicals, not only intraoperatively but also for several hours later. These results indicate that shortening the duration of OLV and avoiding manipulation of lung tissue may inhibit the occurrence of AEIPF resulting from oxygen-derived free radicals.

No drug has been established to decrease the incidence of AEIPF. Few studies reported that steroid, pirfenidone [32], and anticoagulants [33] reduce the occurrence of AEIPF in patients with IPF. However, regarding postoperative AEIPF, so far, no researchers presented a drug that can prevent or decrease the occurrence of postoperative AEIPF. Thus, thoracic surgeons use some drugs reported to reduce the incidence of AEIPF or slow the deterioration of IPF, such as macrolides [34, 35], N-acetylcysteine [36–39], proteinase inhibitor [40, 41], and pirfenidone [42, 43]. The effect of these drugs on decreasing the incidence of postoperative AEIPF remains unclear; therefore, multi-institutional randomized controlled study should be planned in order to determine the effect of these drugs.

### 2.7. Treatment of Postoperative AEIPF

In the absence of any effective therapeutic regimens, postoperative AEIPF seems to be a fatal problem. Although most patients with nonpostoperative AEIPF have been treated with regimens that include high-dose corticosteroids [44], the vast majority shows only partial and temporary improvement. In postoperative AEIPF, the situation is similar. Some investigators have even suggested that mechanical ventilation does not benefit IPF patients presenting with acute respiratory failure [45]. Yokoyama and associates [46] suggest that noninvasive ventilation can be a possible option for the management of acute respiratory failure in patients with AEIPF.

Some studies reported that immunosuppressive agents, such as Cyclosporine A [47–49] and cyclophosphamide [50], improve the prognosis of AEIPF. However, Okamoto et al. [16] reported that patients with AEIPF under methylprednisolone pulse therapy in combination with cyclophosphamide or cyclosporine A did not significantly improve the outcome of AEIPF. In conclusion, there is little evidence that currently accepted treatments are effective in AEIPF, and further studies are needed to clarify the pathogenesis and contribute to the prevention of AEIPF. On the other hand, there are some reports on the effect of hemoperfusion [51, 52] on AEIPF. The study by Seo et al. showed that six patients with AEIPF underwent polymyxin B-immobilized fiber column (PMX) hemoperfusion treatment. In four of six patients, alveolar-arterial difference of oxygen, serum KL-6, and lactate dehydrogenase level were improved after PMX treatment. These four patients were successfully weaned from mechanical ventilation and survived more than 30 days after the initial PMX treatment. However, this study involved a small number of patients and/or the absence of randomization. A randomized controlled study will be more factual.

## 3. Conclusion and Future Directions

AEIPF frequently occurs after lung resection for lung cancer with fatal outcome. Nowadays, there is no definitive treatment guidelines and effective prevention on the occurrence of AEIPF. A randomized study with statistically adequate number of patients is required to identify the etiology, risk factors, prognostic markers, and effective treatments.

## Figures and Tables

**Table 1:** Definition of acute exacerbation of IPF described by Hyzy et al. [10].

Previous or concurrent diagnosis of IPF*	
Unexplained worsening or development of dyspnea within 30 d	
High-resolution CT scan with new bilateral ground-glass abnormality and/or consolidation superimposed on a background reticular or honeycomb pattern consistent with a UIP pattern^†^	
Worsening hypoxemia from a known baseline arterial blood gas^‡^	
No evidence of pulmonary infection by endotracheal aspiration or BAL	
Exclusion of alternative causes, including	
left heart failure	
pulmonary embolism	
identifiable cause of acute lung injury^§^	

*This criterion can be met by the presence of radiologic and/or histopathologic changes consistent with a UIP pattern if a diagnosis of IPF has not been previously established by American Thoracic Society/European Respiratory Society criteria.

^†^Current high-resolution CT scan is acceptable without prior high-resolution CT scan for comparison if none is available.

^‡^Includes evaluation for common bacterial organisms and viral pathogens.

^§^Causes of lung injury include sepsis, aspiration, trauma, transfusion of blood products, pulmonary contusion, fat embolization, drug toxicity, acute pancreatitis, inhalational injury, and cardiopulmonary bypass.

**Table 2:** The incidence and mortality of postoperative acute exacerbation of IPF.

Author	Published year	No. of AEIPF	Incidence of AEIPF (%)	Death of AEIPF	Mortality	Mortality after AEIPF	No. of IPF Pts	Total no. of Pts
Fujimoto et al. [12]	2003	0	0	0	0	NA	21	NA
Kumar et al. [13]	2003	5 (ARDS)	20.8	4	16.7	80	24	988
Chiyo et al. [14]	2003	9	25	3	8.3	33.3	36	931
Koizumi et al. [15]	2004	7	14.9	6	12.8	85.7	47	1103
Okamoto et al. [16]	2004	4	20	3	20	75	20	NA
Kushibe et al. [17]	2007	4	12.1	4	18.2	100	33	1066
Watanabe et al. [18]	2008	4	7.4	4	7.4	100	54	870
Minegishi et al. [19]	2009	8	8.6	3	22.9	37.5	35	NA
Shintani et al. [20]	2010	6	15	5	12.5	83.3	40	1256

AEIPF: acute exacerbation of idiopathic pulmonary fibrosis, NA: not available, Pts: patients, ARDS: acute respiratory distress syndrome.

**Table 3:** The risk predictors of the postoperative acute exacerbation.

Author	Published year	Risk predictors of postoperative AEIPF
Kumar et al. [13]	2003	Low %DLco (AEIPF+ versus AEIPF−; 48% versus 58%), Low %*K*_CO_ (58% versus 70%), High CPI (44 versus 33)
Koizumi et al. [15]	2004	PS > 2, CRP > 2 mg/dL, LDH > 400 IU/L,%TLC < 95%
Okamoto et al. [16]	2004	%VC < 80, LowDLco (value is not described)
Kushibe et al. [17]	2007	%VC < 80%
Watanabe et al. [18]	2008	None
Shintani et al. [20]	2010	%VC (<80.6%) and LDH (≥241 IU/L)

*K*_CO_: levels of carbon monoxide diffusion capacity corrected for alveolar volume;

CPI: composite physiological index; PS: performance status; TLC: total lung capacity;

DLCO: diffusing capacity of the lung for carbon monoxide.
